# Multiple Osteochondritis Dissecans as Main Manifestation of Multiple Epiphyseal Dysplasia Caused by a Novel Cartilage Oligomeric Matrix Protein Pathogenic Variant: A Clinical Report

**DOI:** 10.3390/genes15111490

**Published:** 2024-11-20

**Authors:** Antonio Mazzotti, Elena Artioli, Evelise Brizola, Alice Moroni, Morena Tremosini, Alessia Di Cecco, Salvatore Gallone, Cesare Faldini, Luca Sangiorgi, Maria Gnoli

**Affiliations:** 1 1st Orthopaedics and Traumatologic Clinic, IRCCS Istituto Ortopedico Rizzoli, 40136 Bologna, Italy; antonio.mazzotti@ior.it (A.M.);; 2 Department of Biomedical and Neuromotor Sciences (DIBINEM), Alma Mater Studiorum University of Bologna, 40123 Bologna, Italy; 3 Department Rare Skeletal Disorders, IRCCS Istituto Ortopedico Rizzoli, 40136 Bologna, Italy; evelisebrizola@hotmail.com (E.B.); alice.moroni@ior.it (A.M.); morena.tremosini@ior.it (M.T.); alessia.dicecco@ior.it (A.D.C.); luca.sangiorgi@ior.it (L.S.); maria.gnoli@ior.it (M.G.); 4 Clinic Neurogenetic Neuroscience Department, University of Turin, 10124 Turin, Italy; salvatore.gallone@unito.it

**Keywords:** multiple epiphyseal dysplasia, osteochondritis dissecans, *COMP* gene, pathogenic mutation

## Abstract

Background: Multiple epiphyseal dysplasia (MED) is a clinically and genetically heterogeneous group of skeletal diseases characterized by epiphyseal abnormalities associated with mild short stature. The clinical variability is wide, and the first clinical manifestations still occur in childhood with joint pain and stiffness that evolve into degenerative joint disease. MED, caused by mutations in the Cartilage Oligomeric Matrix Protein (*COMP*) gene, is the most common form of the disease. *COMP*-MED usually shows significant involvement of the capital femoral epiphyses and irregular acetabulum; instead, *COL9A1*-, *COL9A2*-, and *COL9A3*-MED appear to have more severe knee involvement than hips, resulting in a milder presentation than COMP-MED cases. Other complications have been reported, in particular osteochondritis dissecans (OCD), which has been described in two large *COL9A2*-related MED families associated with myopathy. Methods: Here, we report the case of a 24-year-old man affected by COMP-MED with a positive family history for the disease and a clinical presentation that interestingly is characterized by the presence of multiple OCD. Results: To our knowledge, this is the first case of *COMP* mutations related to multiple OCD as the main clinical feature. Conclusions: This report can expand the clinical phenotype related to the pathogenic variants of the *COMP* gene, as it shows that multiple OCD can also be present in *COMP*-related MED as well as in *COL9A2*-related MED.

## 1. Introduction

Multiple epiphyseal dysplasia (MED) is a clinically and genetically heterogeneous group of skeletal diseases characterized by epiphyseal abnormalities associated with mild short stature, chronic joint pain, and stiffness, leading to degenerative joint disease [1,2]. Autosomal dominant MED is related to pathogenic variants in *cartilage oligomeric matrix protein (COMP), matrilin 3 (MATN3), collagen type IX alpha 1 chain (COL9A1), collagen type IX alpha 2 chain (COL9A2), collagen type IX alpha 3 chain (COL9A3)* genes [1,2]. MED caused by *COMP* gene mutations is the most common form of the disease [2,3,4].

*COMP* (cartilage oligomeric matrix protein, OMIM 600310) is a pentameric extracellular matrix protein that catalyzes the assembly of collagens and promotes the formation of well-defined fibrils [1]. It is composed of a coiled-coiled pentamerization domain, epidermal growth factor (EGF)-like or Type 2 (T2) repeats, highly conserved calmodulin (CaM)-like or Type 3 (T3) repeats, and a globular C terminal domain (CTD).

Several pathogenic variants on the *COMP* gene have been correlated to MED, while specific *COMP* mutations have been related to the most severe end of the phenotypic spectrum, known as pseudoachondroplasia (PSACH OMIM 177170). PSACH is a skeletal dysplasia that presents with short limbs, dwarfism, and spondyloepimetaphyseal involvement [1,3]. Most pathogenic *COMP* variants cluster in T3 repeats or exon 15–19 (coding for the CTD); variability of clinical expression has been described, and mutations in the same residues have been related to both MED or PSACH phenotypes [1,3].

In general, in *COMP*-related MED, the first clinical manifestation is pain in the hips and/or knees after exercise occurring in early childhood [3]. Thus, the first clinical evaluation is often performed by pediatricians due to pain, walking delay, waddling gait, knee and finger joint laxity, and/or mild hypotonia [2]. The progression of chronic pain and joint deformity tends to evolve to early-onset osteoarthritis. Additionally, the height may be lower than the expected range as normal for age, resulting in a mild final short stature.

In MED, the clinical variability is wide [3,5]. *COMP*-MED usually shows significant involvement of the capital femoral epiphyses and irregular acetabulum; on the contrary, *COL9A1*-, *COL9A2*-, and *COL9A3*-MED appear to have more severe knee involvement than hips, resulting in a milder presentation compared to *COMP*-MED cases [4,5,6].

Additional manifestations have been reported in MED, as Versteylen and colleagues (1998) described two *COL9A2*-MED families with prevalent osteochondritis dissecans (OCD) [7,8]. Furthermore, familial OCD has been related to pathogenic variants in the *ACAN* gene; this is an autosomal dominant condition with multiple joint OCD, disproportionate short stature, and early osteoarthritis as main clinical-radiological features [9,10]. In contrast to MED, *ACAN* pathogenic variants cause a phenotype characterized by short stature and advanced bone age (OMIM #165800) [1]. In *COMP*-MED, beside affected epiphyses of tubular bones, with typical small rounded phalanges epiphyses, bone age is delayed [1,2,3,4]. Also, minor dysmorphic features (midface hypoplasia, brachydactyly, broad great toes, and lumbar lordosis) can be observed in *ACAN*-related OCD but not in COMP-MED [1]. Lastly, spondylarthrosis has been reported in *ACAN*-related disorders, while in *COMP*-MED the spine is always normal [3].

The term OCD was first coined by König in 1887 to identify osteochondral loose bodies within a joint [11]. OCD (as the delamination of articular cartilage from the underlying subchondral bone) has been reported in different inherited diseases and skeletal dysplasias [11,12]. In OCD cases, the most affected joint is the knee, followed by the ankle, elbow, shoulder, and hip.

The etiology of OCD, prevalent in young populations, is still not well understood. Different theories have been proposed, advocating mechanical, biological, hereditary, and anatomical factors in the pathogenesis of the disease [13,14]. Considering the reports of OCD recurrence in several family members and in twins, with involvement of the knee, capitellum, and talus, it has been suggested that genetic factors may also play a role in the pathogenesis of the disease [14,15].

In this report, we discuss a case of *COMP*-related MED with OCD in multiple sites.

## 2. Case Report

This report was written according to CARE guidelines [16]. The patient was a 24-year-old male who presented to our orthopedics department complaining of left ankle pain and functional impairments during weight-bearing. His height at the time of observation was 162 cm (<3°p) and weighed 68 Kg (25–50°p). He had myopia, and he did not refer to hearing loss.

**Figure 1:**
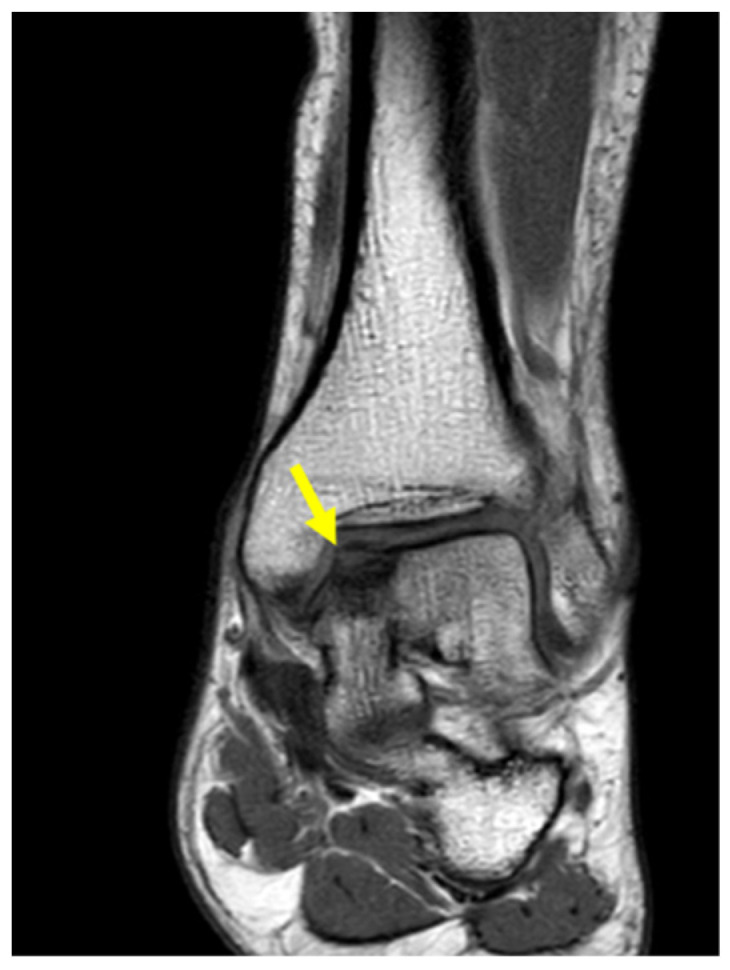
Coronal ankle MRI showing OCD (arrow) on the medial side of the left talus.

His left ankle was slightly swollen, painful to the touch on the antero-medial side. Tenderness was also reported at maximum degrees of ankle flexion and extension. Standard radiographic examinations have been performed, including antero-posterior and lateral weight-bearing views of the ankle, which showed some contour abnormalities, a subtle flattening, and an indistinct radiolucency above the cortical surface of the medial talar dome. Magnetic resonance imaging (MRI) found an OCD of 16 × 10 × 8 mm on the medial side of the left talus (Figure 1).

Two dedicated clinical tools were used to measure clinical outcomes, including pain and function; the American Orthopedic Foot and Ankle Society (AOFAS) scored 67 out of 100, and the visual analog scale (VAS) scored 7 out of 10, respectively.

After taking into account the patient’s functional outcome expectations, surgery was performed on the left ankle. Arthroscopic debridement, sub-endo-chondral retrograde drilling, and implant of bone marrow aspirate concentrate on a hyaluronan scaffold to the OCD were performed [17].

No intraoperative complications occurred. The patient was discharged with an ankle bandage to be maintained for one week. Active ankle mobilization was allowed at the removal of the bandage, and progression to full weight-bearing was allowed only two months after surgery. To expedite functional recovery and enhance clinical outcomes, pulsed electromagnetic fields were applied for two months, starting immediately after surgery. A delayed wound healing at the sinus tarsi level occurred at a one-month follow-up, completely treated through dressings without sequelae.

At six months of follow-up, the patient was able to walk with full weight bearing and without any symptoms. Clinical examination showed complete wound healing and no ankle swelling. A painless full range of motion was allowed. AOFAS and VAS scores improved from 67 to 85 and from 7 to 2, respectively.

### 2.1. Past Medical History

Neurodevelopment was normal. After an accidental trauma of the head at the age of 18 months, the patient suffered seizures. He had another episode at 6 years old, and the electroencephalogram (EEG) showed some abnormalities; thus, he was treated with antiepileptic therapy for two years. No brain abnormalities were detected by MRI. Lower limb X-rays at 4 years old showed mild alteration in the proximal femoral epiphysis. The patient also reported having pes planus in childhood and that surgery correction was proposed, but it was refused. No further clinical evaluations were performed in childhood or adolescence, and no other X-rays were available.

**Figure 2:**
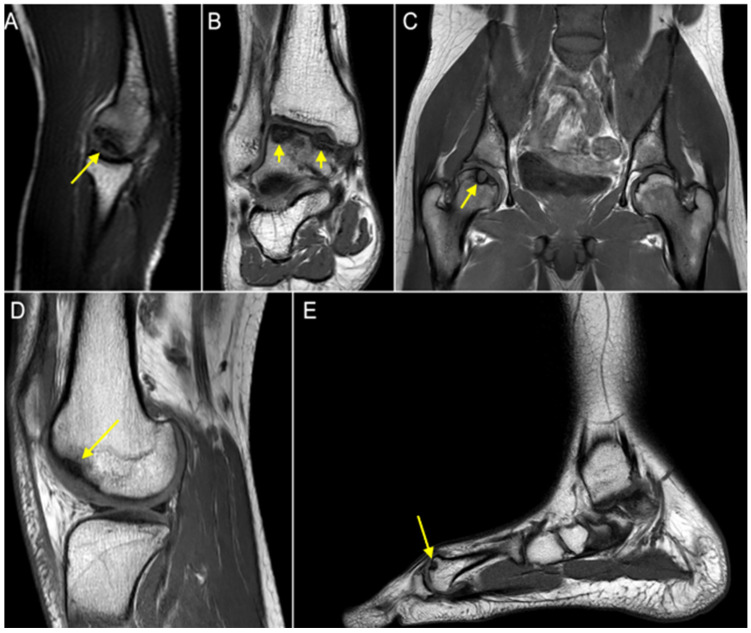
(**A**) OCD of the capitellum; (**B**) OCD of the right talus; (**C**) OCD of the right hip; (**D**) OCD of the right knee; (**E**) OCD of the left first metatarsal. Arrows indicate OCD sites.

When he was 20 years old, a radiological evaluation due to right elbow pain revealed OCD of the capitellum (Figure 2A). Further imaging investigation showed OCD also at the ankles, bilaterally. At the age of 21 years, he had his right ankle treated arthroscopically with symptom resolution (Figure 2B). One year later, he underwent a right hip core decompression for OCD (Figure 2C), referring to temporary benefit after the surgery. In addition, the patient was assessed by a total-body MRI, which showed asymptomatic distal right femur (Figure 2D) and left first metatarsal OCD (Figure 2E).

To summarize, at 21 years old, the patient had multiple OCD diagnoses of the right elbow, right distal femur, right hip, bilateral ankles, and left first metatarsal.

At 24 years old, the patients received a molecular diagnosis of *COMP*-related MED.

**Figure 3:**
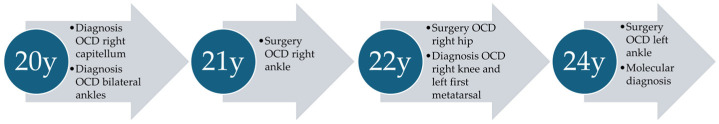
Timeline of the patient’s diagnostic and treatment.

A timeline of the patient’s diagnostic assessments and treatment interventions is presented in Figure 3.

### 2.2. Family History

**Figure 4:**
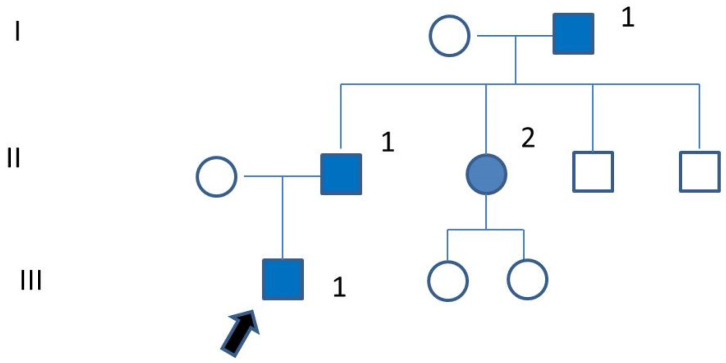
Family pedigree. III-1: proband (black arrow); II-1: short stature, joint pain with muscle weakness at the age of 3 years, bilateral hip arthroplasty (<50 years). II-2: short stature, early onset multi-joint pain, X-rays in childhood with epiphyseal abnormalities (referred). I-1: significant short stature, multi-joint pain. Affected individuals are in blue.

The patient had a positive family history of MED (see pedigree in Figure 4); the clinical diagnosis was made in the family, but there was no genetic confirmation. His father, grandfather, and paternal aunt experienced multi-joint pain and underwent joint replacements at an early adult age.

We collected clinical data and performed clinical examinations on the patient’s father, now a 67-year-old man. He is 160 cm tall (<3°p); his weight is 67 Kg (25–50°p). Clinical reports about his childhood describe that he had joint pain with muscle weakness at the age of 3 years. At the age of 6 years, he was 116 cm tall; he showed short hands and mild varus deformity of the knee. He underwent bilateral hip arthroplasty at ages 47 and 48. He also underwent hemithyroidectomy and inguinal hernia surgery. He had myopia of the left eye treated with laser therapy. Recently, he was evaluated for surgical treatment of both shoulders.

The other affected members of the family presented short stature (the grandfather was 150 cm tall), limited range of motion at many joints, genu valgum, and kyphoscoliosis.

### 2.3. Molecular Findings

Molecular analysis of 442 skeletal dysplasia-related genes (including *COMP*, *COL9A1*, *COL9A2*, *COL9A3*, *MATN3*, and *ACAN* genes) by the next-generation sequencing (NGS) panel was performed in the proband.

The establishment of the nucleotide libraries necessary for sequencing was carried out using the SureSelect All Exon V6 kit (Agilent, Santa Clara, CA, USA) following the extraction of the DNA from the patient’s blood. Sequencing of DNA libraries was performed using NGS (100 bp-paired end reads) technology. The bioinformatics analysis provided for the alignment of the sequences to the reference genome (GRCh37/hg19) with the BWA algorithm, the removal of the sequences with specific alignments, the calling of the genetic variants, and the coverage and coverage depth control for the analyzed genes within the required genetic panel.

The detected genetic variant was subjected to resequencing using Sanger technology with the design and synthesis of synthetic oligonucleotides for the selection and PCR amplification of small gene portions containing the mutation found. The PCR product was later sequenced by Big Dye Terminator V 3.1 cycle sequencing (Applied Biosystem, Waltham, MA, USA) and capillary electrophoresis on an automatic sequencer ABI3100avant (Applied Biosystem).

The interpretation of the genetic variants was performed according to the guidelines set by the American College of Medical Genetics (ACMG 2015). Genetic variants that have been detected within the required genetic panel are annotated by consulting population databases (Exome Aggregation Consortium, 1000 Genome Database, Exome Variant Server, and GnomAD) and clinical genetic databases (OMIM, ClinVar, HGMD, and GWAS). Furthermore, each variant is subjected to in silico evaluation of the possible effects that may affect the structure and/or function of the protein (Polyphen2, SIFT, CADD Mutation Taster, and Mutation Assessor) and of the conservation profiles of the same (PhyloP).

**Figure 5:**
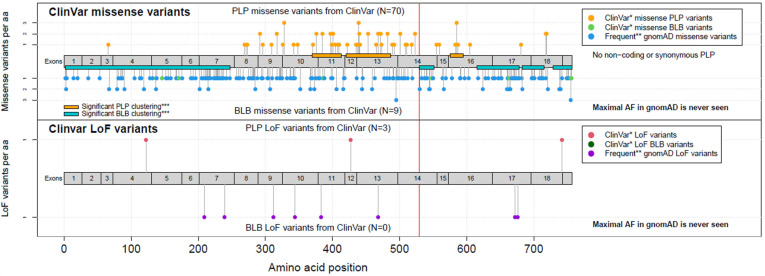
*COMP* missense (above) and loss of function (below) variant distribution from Clinvar. The vertical red line indicates this report variant’s location. PLP: pathogenic or likely pathogenic; BLB: benign or likely benign. * ClinVar version of 09.01.2022. ** with AF > max (AF of PLPs). *** Clusters version 21.11.2020.

**Figure 6:**
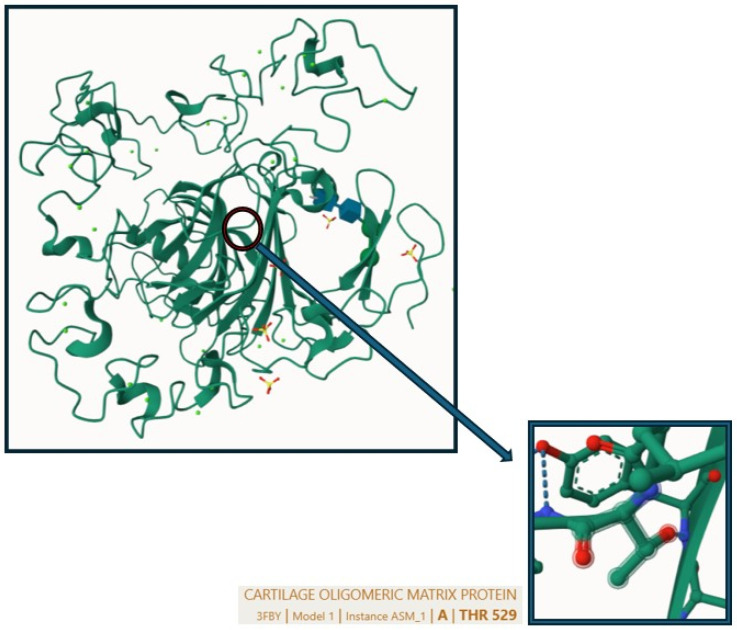
3D representation of the COMP protein structure through the AlphaFold method. The region of the Thr529 residue is highlighted in the box.

We identified the heterozygous variant c.1586C>A, p.(Thr529Asn) in exon 14 of the *COMP* gene (NM_000095.2) (Figure 5). The variant found can be interpreted as pathogenic according to the guidelines of the American College of Medical Genetics (ACMG, 2015), as it is located in a gene known to cause multiple epiphyseal dysplasia (MED). The amino acid substitution (Thr529Asn) may adversely affect the function of the COMP protein, which is critical for cartilage matrix formation, supporting the idea that this variant alters a critical functional part of the protein and the patient presents a phenotype compatible with MED, thus satisfying both direct evidence of pathogenicity and indirect evidence based on the functional impact the substitution has on the structure of the protein (Figure 6). Moreover, the variant has been reported as likely pathogenic in Clinvar and is absent from population databases.

Sequence analysis for the identified *COMP* variant revealed the same variant in the patient’s father.

## 3. Discussion

In this report, we described an interesting case of a young adult man affected by MED due to a novel *COMP* gene variant associated with multiple OCD in several joints. The accurate diagnosis of a *COMP*-related disorder in his case could not be made straightforward, especially without knowing the family history information. The multidisciplinary approach and the great experience in rare bone diseases of the involved team of professionals facilitated the diagnostic procedure and benefitted the patient and the family in the management, treatment, and information on the risk of recurrence of the disease.

In MED, hip and knee joint pain are usually the first clinical signs that occur during childhood, but they are not specific to the disease. Radiographic evaluations performed during childhood can reveal a pattern of ossification and distinctive features, such as epiphyseal abnormalities at different sites, that can distinguish the various forms of MED. These abnormalities result in early-onset osteoarthritis in multiple joints, which may be seen as early as the end of skeletal growth and may require joint replacements at a young age.

In this case, the diagnosis of MED was missed during childhood, and the clinical and radiological signs, together with a positive family history, are key points to lead to an early diagnosis. Elements such as early-onset osteoarthritis, parents’ early-age joint replacements, and radiographic abnormalities can all be put together into the patient’s medical history and help address the questions raised in the clinical context. Nevertheless, the case here presented only came to the clinicians’ attention at the age of 18 years old, mainly due to the OCD in multiple sites. The unavailability of the radiographic performed in childhood, in particular of the knees and of the carpal bones, did not permit an early radiological diagnosis of the disease.

In addition, the performed molecular analysis identified a novel *COMP* pathogenic variant, confirming the clinical diagnosis. The type of the identified mutation and the site of the mutation (type III repeat, 8, C-type motif) are typical of MED; in fact, most of the variants have been found in type III repeats [3,18].

OCD is not a main characteristic of *COMP*-related MED. Instead, in two *COL9A2*-related MED families, several individuals affected with OCD have been reported [7]. This report can expand the clinical phenotype related to the pathogenic variants of the *COMP* gene, as it shows that multiple OCD can also be present in *COMP*-related MED as well as in *COL9A2*-related MED.

*COMP* pathogenic variants are causative of phenotypes that represent a continuum clinical spectrum, with an overlap between MED and mild PSACH. A few genotype-phenotype correlations have been observed, and variants in the same aminoacidic residues have been related to both MED and PSACH.

Moreover, different aminoacidic changes in Threonine 529 were reported as pathogenic/likely pathogenic in MED/PSACH patients in mutational databases [18,19]. Interestingly, mutations in type III repeats 6 to 8 are generally associated with PSACH [3,18]. In contrast to this genotype-phenotype correlation, the reported patient was not affected by PSACH, consistent with the clinical expression variability of the disease and the fact that mutations in the same residues could cause both phenotypes.

Neurological evaluation also should be considered in the clinical follow-up of patients with *COMP*-MED and *COL9A*-MED; even if the patient described here had no neurological alteration, myopathic signs have already been previously reported in the medical literature [6].

The mechanisms involved in clinical expression and the phenotypic variability are not yet fully understood. Indeed, studies on cell and mouse models have suggested that endoplasmic reticulum (ER) stress due to misfolded protein storage is the basis of the disease’s pathogenic mechanism [20,21]. In vitro and in vivo studies on a specific *COMP* mutation, in fact, revealed protein misfolding that causes its retention in chondrocytes’ rough endoplasmic reticulum (rER), with co-retention of other ECM proteins, including type IX collagen and aggrecan (seconded by the *ACAN* gene) [20]. It has been suggested that the mutation in the *ACAN* gene involving the domain that mediates interactions with other proteins in the ECM might ossiculate these interactions and might contribute to a disorganized ECM in the growth plate [9]. Therefore, it can be supposed that OCD in both *ACAN*-related disorders and *COMP*-MED could be due to similar molecular mechanisms. However, not all studies confirm this hypothesis, and it is not clear how the same mutations can be related to both mild and severe phenotypes; further investigations should be made to elucidate these aspects.

As said, OCD has already been reported in *COL9A2* MED; *COL9A2* mutations cluster in specific regions, and the pathogenic mechanism in *COL9*-MED is poorly understood; however, it seems to be different from that in *COMP*-MED as it affects the composition, structure, and function of the cartilage extracellular matrix and does not cause ER stress [20,21]. This effect (disrupted ECM composition) seems more similar to the supposed pathogenesis in *ACAN*-related disorders [21] than in *COMP* MED, not explaining the OCD presentation of our patient.

Further studies (i.e., mouse models with specific *COMP* or *ACAN* mutation, expression studies, and biochemical investigation in cartilage or muscle samples) are needed to clarify the mechanisms leading to the clinical variability of MED and the association of very particular clinical characteristics such as OCD or myopathic signs. As with other skeletal diseases, increasing knowledge about disease pathogenesis may lead to new targeted therapies [21,22]. According to ER stress-mediated mechanisms, treatments that target active autophagy against protein retention may be a promising approach, as evaluated in a recent study [23].

In addition, in the light of this report, in the presence of OCD in multiple sites without extra-skeletal involvement suggesting other genetic conditions, MED must also be considered for differential diagnosis and not only an *ACAN*-related disorder.

Immediately after the detection of OCD in one anatomical site, especially in the presence of short stature and/or positive family history, a genetic predisposing condition to multiple OCD or genetic skeletal dysplasia needs to be evaluated. Furthermore, in a suspected case of multiple/familial OCD, an MRI evaluation in diverse sites should be performed to try to identify other lesions, as proposed by Takeda and colleagues (2021) [24].

The findings presented in this study should be interpreted in light of certain limitations. First, as a case report focusing on a single patient, the ability to generalize these results to a broader population is limited. Unfortunately, not all affected family members were available to perform molecular confirmation of the diagnosis, but clinical and radiological data of the family members were in line with the diagnosis of autosomal dominant MED and with the well-described clinical variability of the disorder. Lastly, other genetic, environmental, or lifestyle factors may have acted as confounders, complicating efforts to isolate the effects of the specific genetic variation identified in this patient.

## 4. Conclusions

In conclusion, the presence of multiple OCDs could lead to the suspicion of a genetic condition. The diagnosis of a genetic disease has multiple implications for patient clinical management (i.e., surveillance and treatment) and for the patient’s family. In fact, molecular tests can be offered to pursue the diagnosis, and the risk of recurrence can be established. Molecular analysis accelerates the diagnostic pathway, even before the first clinical manifestation, enabling early diagnosis and management, thus preventing or reducing disease complications and providing the best-targeted treatment and multidisciplinary follow-up to these individuals.

## Data Availability

The data presented in this study are available on request from the corresponding author due to privacy, legal, and ethical reasons.
